# A Review of Type III Gastroesophageal Junction Adenocarcinoma and the Importance of Early Detection of Gastric Cancer

**DOI:** 10.7759/cureus.59917

**Published:** 2024-05-08

**Authors:** Alisha Jadhav, Mumen Ayyat, Francesco M Serafini

**Affiliations:** 1 Surgery, St. George's University School of Medicine, Brooklyn, USA; 2 Surgery, Brookdale University Hospital Medical Center, Brooklyn, USA; 3 General Surgery, Brookdale University Hospital Medical Center, Brooklyn, USA

**Keywords:** preoperative chemotherapy, gastric cancer screening, gi bleed, gastroadenocarcinoma, gastroesophageal tumor

## Abstract

Gastric adenocarcinoma is the most common type of gastric cancer in the United States. Multiple factors can predispose a patient to develop such a malignancy, including having a history of *Helicobacter pylori* infection, tobacco use, alcohol use, specific genetic mutations, and being of Asian or Hispanic descent. Surgery is currently the only curative treatment for localized disease. With metastatic disease, the rate of survival decreases significantly, and most often, the only treatment option is palliative chemotherapy with or without combination radiation therapy. In the case of a 58-year-old man diagnosed with a gastroesophageal junction type III gastric adenocarcinoma that extended into the distal esophagus, what was thought to be a resectable tumor had already invaded vital neighboring organs, therefore, we were unable to eradicate the disease from this patient.

## Introduction

A gastroesophageal junction (GEJ) mass can be indicative of a malignant lesion. Gastric cancer can be deadly if not treated quickly and effectively. Identifying the cancer at an early stage is crucial to improving the rate of survival in affected patients. At this point, there is no prophylactic screening in the United States for gastric cancers [1]. Diagnosis of gastric malignancies usually only occurs after patients are symptomatic, presenting with manifestations such as abdominal pain, anemia, and weight loss, or because of an incidental finding on imaging. Histological classification of the gastric tumor cells via endoscopic biopsy is required to confirm the diagnosis of gastric cancer and specify the type of lesion, such as gastric adenocarcinoma or gastrointestinal stromal tumor (GIST). The overall survival of advanced gastric adenocarcinoma, once a diagnosis has been confirmed, is only three to five months without any interventions; therefore, treatment is essential. Surgical resection of the mass following or followed by chemotherapy provides the most prolonged survival rate in patients with gastric cancer.

An innovative study called the Medical Research Council Adjuvant Gastric Infusional Chemotherapy trial, also known as the MAGIC trial, evaluated the effects of preoperative chemotherapy on gastric, esophageal, or GEJ tumor regression. The study showed that the overall survival of patients with adenocarcinoma of the stomach or esophagus is significantly improved when compared to that of patients who underwent surgery alone [2]. This trial set a precedent for a new method of increasing the effectiveness of treating gastric cancers. Chemotherapy on its own can also be palliative for patients who have unresectable or recurrent tumors [3].

There are three types of GEJ tumors: the Siewert classification of a GEJ mass is based on the location of the epicenter of the tumors and its relationship with the GEJ. Type I lesions are located 5 cm proximal to the GEJ, type II lesions span the GEJ and have their focal point up to 2 cm below the GEJ, and type III cardiac tumors extend up to 5 cm into the stomach [4]. It is important to differentiate between the three to have optimal surgical planning and specified preoperative and postoperative care [5]. One of the most common procedures for the removal of a gastroesophageal mass is an Ivor Lewis esophagectomy, which involves a laparotomy and a right thoracotomy for the resection of the tumor [6].

We present a case of a 58-year-old Hispanic male presenting with anemia, dizziness, and emesis, the plan was to do such a procedure to resect a type III GEJ gastric adenocarcinoma that was spanning from the distal esophagus to the cardia of the stomach using the Ivor Lewis esophagectomy technique. When the mass was identified at the time of surgery, it was evident that the tumor had already started invading the surrounding structures, including the diaphragm and the aorta.

## Case presentation

**Figure 1:**
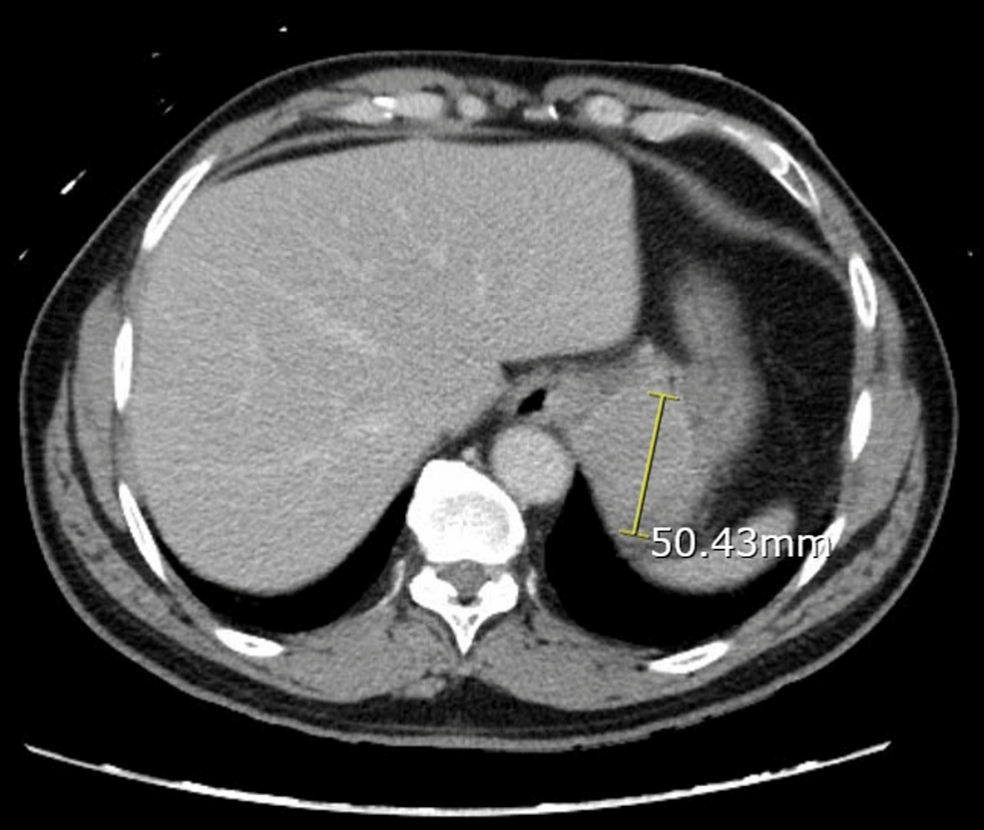
CT of the abdomen and pelvis (coronal section) demonstrating a 5 cm solid mass in the gastric cardia.

**Figure 2:**
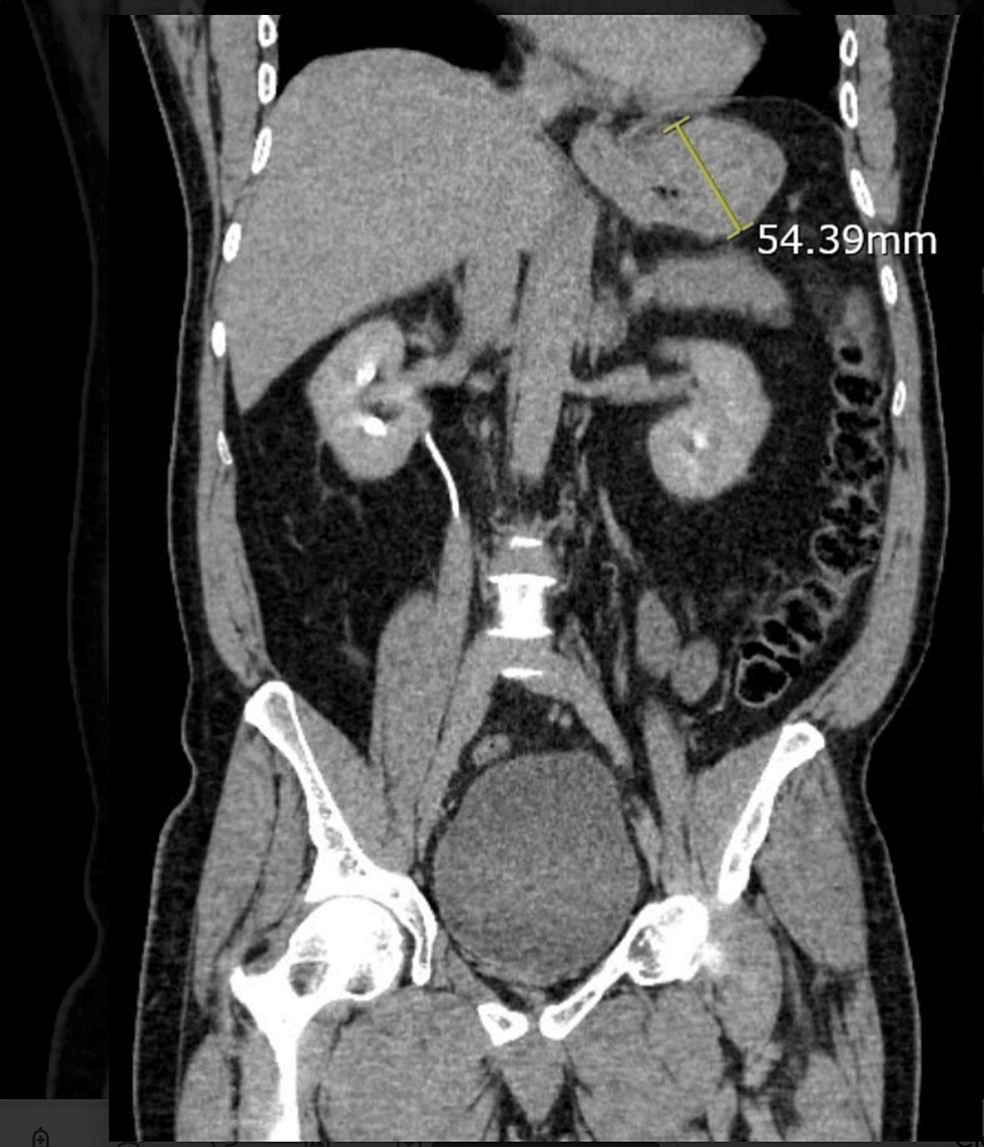
CT of the abdomen and pelvis (coronal section) demonstrating a 5 cm solid mass in the gastric cardia with distal esophageal involvement.

A 58-year-old male with a past medical history of hypertension, iron-deficiency anemia, and type II diabetes mellitus presented to the ED in August of 2023 with dizziness, vomiting, and anorexia. He also stated that he lost 7 lbs in the past 10 days. The patient denied any significant family medical history. His initial hemoglobin level on admission was 7.8 g/dL. The patient continued to bleed and required blood transfusions. A computed tomography (CT) scan of his chest, abdomen, and pelvis demonstrated a 5 cm solid mass in the gastric cardia with distal esophageal involvement (Figures 1, 2).

An esophagogastroduodenoscopy (EGD) was done four days after admission that confirmed the presence of a 49.2 x 37.9 mm gastric cardiac mass infiltrating the gastroesophageal junction and distal portion of the esophagus, most likely arising from the mucosa extending into the muscularis propria, with celiac lymphadenopathy and multiple malignant lymph nodes. A biopsy of the lesion came back positive for a poorly differentiated gastric adenocarcinoma, intestinal type with ulceration, and fragments of squamous mucosa with distal esophageal involvement. The patient was discharged with outpatient follow-up but returned to the emergency department the following day with an episode of syncope while eating breakfast. A CT scan of his chest was done on admission and showed an acute occlusive pulmonary embolism. The use of anticoagulants was contraindicated due to the risk of heavy bleeding in our patient. This necessitated the placement of an inferior vena cava filter. A subcutaneous infusion port was also placed and the patient underwent four cycles of FLOT (fluorouracil, leucovorin, oxaliplatin, and docetaxel) chemotherapy.

The patient was then referred to surgical oncology planning for the resection of the gastric adenocarcinoma, which was to be followed by adjuvant chemotherapy. The plan was made for a robot-assisted Ivor Lewis esophagogastrectomy. During the open exploratory laparotomy, it was quickly found that the stomach adhered to the aorta posteriorly and to the diaphragm. Unable to identify a clear delineation between the aorta and the stomach and safely resect the tumor, the procedure was abandoned. The patient was discharged five days after the operation, and he is currently undergoing combination chemoradiation, with the possibility of surgery after completion of the combination therapy.

## Discussion

In our patient, the risk of damaging vital structures was too great to proceed with the removal of the tumor. When the disease process gets to this advanced stage, there are not many viable options other than palliative chemotherapy. Our patient presented with alarm symptoms, such as anemia secondary to bleeding, weight loss, and fatigue, indicating that his disease is in the advanced stage. The patient does not have a family history of gastric cancer, nor has he presented to the emergency department or his primary care physician with alarm symptoms in the past. The fact that the cancer was diagnosed at a later stage in this disease process is a compelling reason why the cancer was able to invade to the extent that it did.

Ideally, early identification and removal via endoscopy (in cases of type I GEJ tumor) or simple gastrectomy would be the optimal way to treat and eradicate the disease effectively. Gastric cancers are responsible for 783,000 deaths per year, making it the third most deadly cancer among men in the world [7]. Screening and early detection of gastric carcinoma can extend the lives of affected patients in the future. Xia and Aadam (2022) [8] discuss a retrospective cohort study from 2008 to 2014 done in California, which demonstrated that ethnicity does play a factor when it comes to the incidence of gastric cancer. Those of Asian, Hispanic, and black populations have a 50% increased risk for gastric cancer when compared with the non-Hispanic white population.

According to Xia and Aadam, patients with early-stage disease who have resectable tumors with negative margins have a lower burden of circulating tumor DNA (ctDNA) and fewer alterations in the DNA itself. Compared to commonly tested tumor markers such as carcinoembryonic antigen (CEA) and carbohydrate antigen 19-9 (CA 19-9), these newer biomarkers offer more sensitivity and specificity. Long-noncoding RNA and circular RNA levels in serum can also be used to detect the presence of early gastric cancer and to monitor the extent of invasion of gastric cancer and if there is a presence of lymphatic metastasis. Incorporating these biomarkers as screening tools for gastric cancers in at-risk patients could be beneficial to improving the early detection of the disease. Overall, further research is required to assess the practicality of these markers in cancer screening but they provide a potential future direction for advancements in early gastric cancer detection [8].

The same report mentioned that under-experienced endoscopists have been known to miss precursor lesions and gastric cancers. More advanced endoscopic training and artificial intelligence (AI) advancements can improve the detection rate of malignancies. Xia and Aadam discuss how AI has already been used to detect gastric polyps, predict Barrett's esophageal metaplasia, and improve endoscopic technology. In China, a novel AI system called ENDOANGEL-ID has been programmed to learn retrospective and real-time endoscopic images from over 100,000 patients. It has demonstrated increased sensitivity and improved early detection of gastric cancer in trials. Another AI system in Korea called AI-scope has been shown to identify gastric lesions and measure the depth of invasion. This method has proved to be superior to endoscopic ultrasound. Implementing this technology can be expensive and requires further resting, but it can provide a foundation for improved screening of gastric cancer [8].

The early detection of gastric cancer requires financial and community support; however, a study by Saumoy et al. in 2018 [9] suggested that endoscopic screening for gastric cancer in high-risk racial and ethnic groups in the United States at the beginning at the age of 50 years with a surveillance every three years is cost-effective. In Japan, where the incidence of gastric cancer is high, screening with double-contrast radiographs with photofluorography for gastric cancer is recommended for patients older than 50 years every year. This practice has been in effect since 1960. The five-year survival rate is 15-30% better in those who are screened for the disease versus those who are diagnosed because of symptom presentation. Just as there is a well-known concrete method for colorectal cancer screening, perhaps a new multi-disciplinary model for gastric cancer screening is needed, especially for high-risk populations and ethnic groups [10].

## Conclusions

The overall survival rate of those inflicted with advanced gastric adenocarcinoma is low. In this report, we discussed the case of a 58-year-old male patient who had a gastric adenocarcinoma that invaded too far into the posterior aspect of the aorta to resect safely without damaging the neighboring vital organs. Given the advanced stage of his disease, there are not many more options other than a combination of chemotherapy and radiation with the hope of downstaging the tumor to make it amenable to surgical resection. We can hope that early screening methods, such as prophylactic endoscopy screening every few years or testing for more specific biomarkers for gastric cancers, can lead to increased detection, treatment, and survival of those affected by gastric cancer. By incorporating some of the methods of screening that countries such as Japan and Korea use, we can hope to avoid such advanced disease processes as the one presented in this report. This case had an undesirable outcome, but it shows us the importance and the need for early detection and incorporation of screening for gastric cancer in our medical practice.
